# The effects of dune plant roots on loggerhead turtle (*Caretta caretta*) nest success

**DOI:** 10.1002/ece3.11207

**Published:** 2024-04-01

**Authors:** Olivia T. Redding, Max C. N. Castorani, Jake Lasala

**Affiliations:** ^1^ Department of Environmental Sciences University of Virginia Charlottesville Virginia USA; ^2^ Sea Turtle Conservation and Research Program Mote Marine Laboratory Sarasota Florida USA

**Keywords:** dune, loggerhead, nest, predation, roots

## Abstract

Sand dunes are supported by the extensive root systems of dune plants that anchor the dune and protect it from erosion. While all plants that grow on the dunes support their structure, invasive plants can outcompete the native and non‐native dune plants for resources such as nutrients, sunlight, and space to grow. During the summer, sea turtles lay nests on beaches and near dunes; however, their eggs and hatchlings are at risk of destruction and entrapment by dune plant root penetration. Dune plant roots can penetrate sea turtle nest cavities, thus decreasing hatch success of the eggs and emergence success of the hatchlings. We aimed to determine how plant roots impact threatened loggerhead sea turtle (*Caretta caretta*) nests on Casey Key, Sarasota County, Florida, USA and to assess the factors affecting plant root invasion. Specifically, we determined the effect of plant roots on loggerhead sea turtle nest success, the extent of the impact of invasive plants over non‐invasive plants on nests, and if the distance from the dune (barrier) affects whether roots will penetrate the nest. From July to August 2022, we excavated 93 nests to determine the extent of root penetration and identify associated plant species. This field campaign was supported by a long‐term dataset (1987–2022) on loggerhead sea turtle nesting across the region. We found that root presence decreased hatch success by 21% and emergence success by 18%, compared to nests that lacked roots within the nest chamber. Nests closer to the dune were more likely to have a higher proportion of root damage and lower hatch and emergence success. This study helps advance understanding of how native and non‐native plants affect sea turtle reproductive success and helps inform coastal management aimed at conserving threatened loggerhead populations.

## Graphical abstract

Dune plants are important resources for providing structure to dune ecosystems and habitat for many species that live in dunes. However, they can be detrimental to the hatch and emergence success of loggerhead sea turtle hatchlings by invading and encasing eggs and trapping hatchlings in the nest cavity.

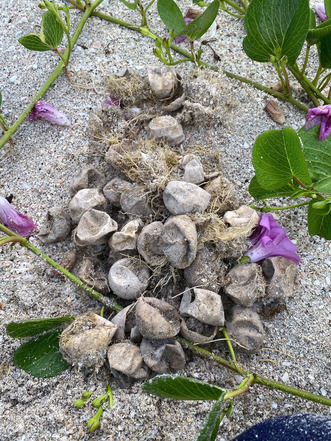

## 1 INTRODUCTION

Dunes are ecologically and economically valuable coastal ecosystems that protect adjacent residential and commercial areas from storms, flooding, and other events that can cause damage to coastal towns and cities (Richardson & Nicholls, 2021). Due to their important functions, fostering strong dunes that are secure and structurally sound is crucial for coastal managers. There are many ways to support dune structure, but among the most sustainable and natural methods is by using vegetation (Stockton, 2023). Many varieties of plants grow on dunes, including those with extensive root systems that anchor the sand and prevent erosion, encouraging dune accretion and plant growth and spread (Conrad et al., 2011). Dunes also create an irreplaceable habitat for many animals (Sigren et al., 2014), such as ghost crabs (Schlacher et al., 2011), sand mice (Stoddard et al., 2019), and a nesting habitat for shore birds (Costa et al., 2023) and sea turtles (Bolten & Witherington, 2003).

**FIGURE 1:**
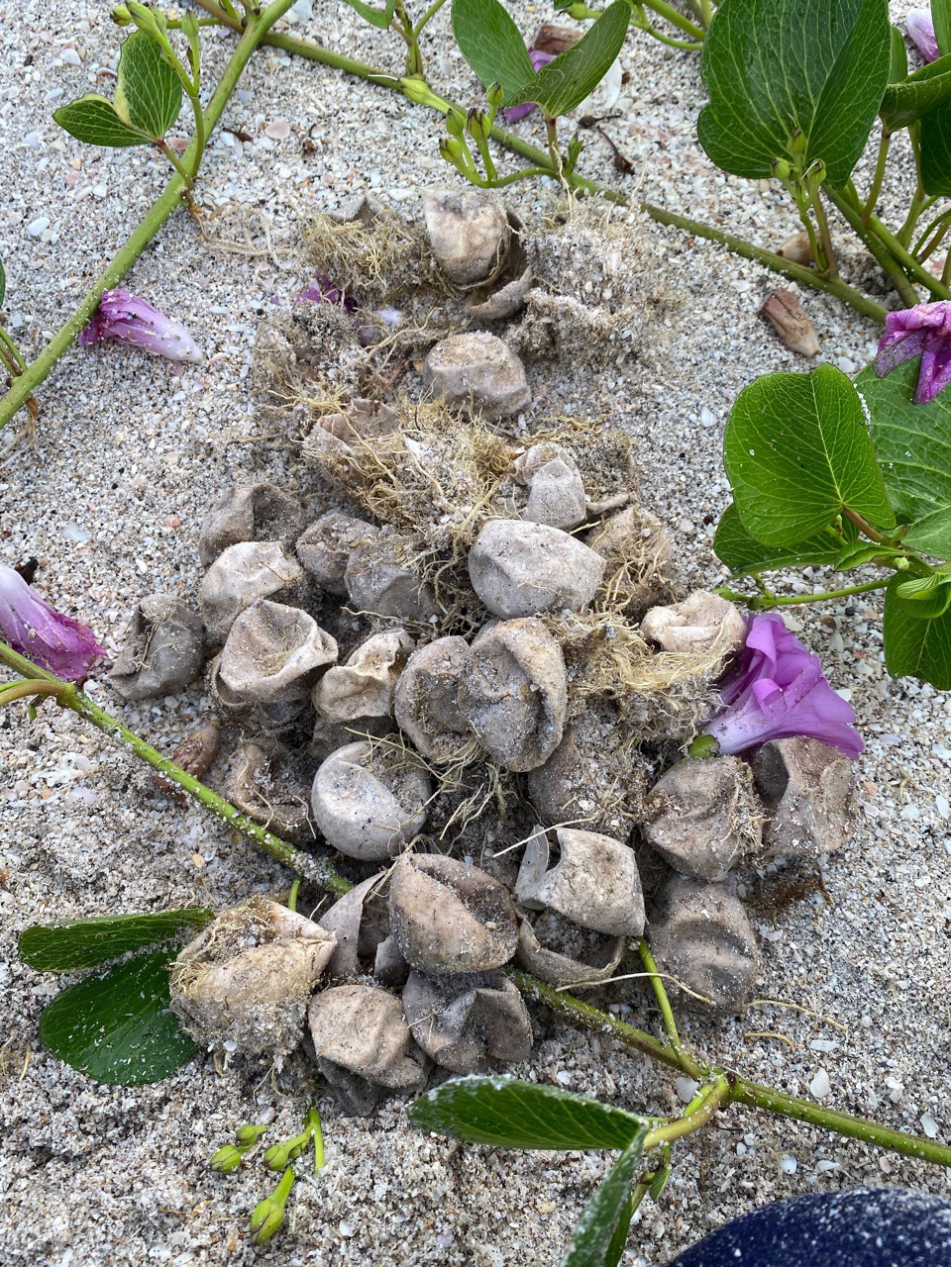
Roots encasing and invading loggerhead sea turtle eggs surrounded by railroad vine (*Ipomoea pes‐caprae*) (cover image: by K. Mazzarella).

Dune plants include native and non‐native species that provide shelter and food for other species (Ewel et al., 1999) and stabilize the dune (Charbonneau et al., 2016). Native plant species are defined as those that naturally grow in a specific environment; in our study, the Gulf Coast of Florida, USA, sea oats (*Uniola paniculata*) and railroad vine (*Ipomoea pes‐caprae*) are prominent (Figure 1). Non‐native plants are defined as species that are introduced to the region of study; examples from our study include beach naupaka (*Scaevola taccada*). Invasive plants are defined as plant species that establish outside of their normal geographic range, similar to non‐native species, but that outcompete other species in the same area for resources and space (Cardinale et al., 2019). Invasive plant species can have direct negative consequences for native plants and can disrupt ecosystem function, as they often lack natural predators or pathogens in their invaded ecosystems (Hiatt et al., 2019). Prominent examples of invasive coastal plant species in Florida include the Australian pine (*Casuarina glauca*) and umbrella tree (*Schefflera actinophylla*) (Williams, 2007). Florida, where this study was conducted, has a high proportion of endemic taxa that contributes to global species richness; it is the third state in the United States, behind Hawaii and California (USFWS, 2017), with the most numerous threatened and endangered species of plants and animals (Hiatt et al., 2019). Florida dune ecosystems also harbor 30 plant and animal species that are considered rare within the state (FDEP, 2024). It is important to protect native plants and animals from invasive species (Pyšek et al., 2012) without compromising dune ecosystem function and biodiversity (Hiatt et al., 2019).

Loggerhead sea turtles (*Caretta caretta*) are prolific nesters that lay 1–7 clutches during the nesting season about every 14 days (Bolten & Witherington, 2003). Female loggerhead turtles have a remigration period of 1–9 years, depending on resource availability and quality (Bolten & Witherington, 2003). In Florida, USA, loggerhead clutches contain an average of 112 eggs (Bolten & Witherington, 2003) and sea turtles lay thousands of eggs annually on Florida beaches each nesting season, which lasts from March to October (FWC, 2022). Sea turtles can choose where to nest and frequently lay their nests in the upper portion of the beach (Lasala et al., 2023).

Dunes are nutrient‐poor ecosystems and lack reliable sources of freshwater, creating challenges for dune plants that are vital for coastal erosion protection (Hannan et al., 2007). Sea turtles are a critical species in transporting nutrients between marine and terrestrial environments in the form of eggs (Bolten & Witherington, 2003). In 2020, over 49,000 loggerhead nests were observed on Florida's 27 core index beaches (FWC, 2022), allowing these beaches to become rich in nutrients from a wide variety of marine foraging grounds (Bouchard & Bjorndal, 2000). Nutrients from sea turtle nests are dispersed throughout the terrestrial environment and sea turtles bring nutrients from the beach back to the marine environment. In the terrestrial ecosystem, nutrients from egg membranes and shells from hatched hatchlings, as well as eggs in various developmental stages that failed to hatch, remain in the nest chamber and are dispersed through the ecosystem by decomposers, predators, and plant roots that penetrate the nest (Bouchard & Bjorndal, 2000). Less than a third of the nutrients from the eggs return to the marine environment as hatchlings; thus, the large majority remains in the terrestrial ecosystem (Bolten & Witherington, 2003). Sea turtle clutches also provide a source of freshwater to dune plants; eggs absorb water from their environment early in development and can contain high amounts of water due to their permeable shells and the lower water potential inside the egg (−950 kPa; Wallace et al., 2006) compared to the sand environment (−5 to −50 kPa; Ackerman, 1997). By providing resources that encourage root growth by dune plants; egg, and hatchling nutrients function to stabilize the dune ecosystem and prevent erosion, in turn, supporting sea turtle nesting habitats (Hannan et al., 2007). Nesting females are largely influenced by beach characteristics when selecting a nesting site, including the presence of vegetation, as some species preferentially nest in areas with higher vegetation density (Guerra et al., 2021).

**FIGURE 2:**
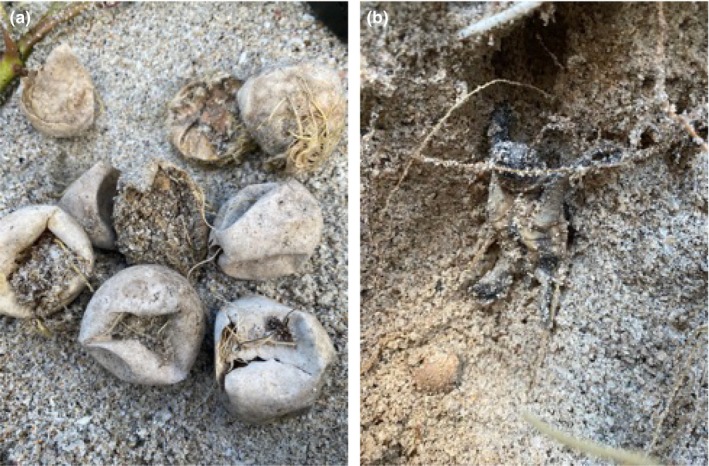
(a) Loggerhead sea turtle eggs with root invasion. (b) Loggerhead hatchling trapped by roots in the nest chamber wall.

However, sea turtle populations are in a precarious position across the world; out of the seven extant species, the six species found in the United States are listed by the International Union for the Conservation of Nature (IUCN) as vulnerable to critically endangered (IUCN Red List, 2022). Threats to sea turtles include climate change, coastal development, fishing, and predation (Bolten & Witherington, 2003), including by dune vegetation. While sea turtles are beneficial for dune nutrients, dune plants can be detrimental to sea turtle nest success. As roots seek out nutrients, they can penetrate sea turtle eggs and arrest embryo development (Conrad et al., 2011). These roots can restrict hatchlings from emerging from the egg by encasing the shell and long‐reaching roots can trap hatchlings within the nest chamber (Conrad et al., 2011; Staines et al., 2019). Researchers have suggested that greater amounts of vegetation may lead to higher organic matter (humus) and fungal load in the sand, increasing the chances of infection and embryonic death (Staines et al., 2019). Hatchlings become trapped in the nest as they attempt to reach the surface by roots that crisscross the nest chamber itself, as well as the walls of the chamber (Staines et al., 2019). These factors can decrease nest hatch success and hatchlings trapped by the roots can decrease their likelihood of emergence (Figure 2) (Shaver et al., 2020; Staines et al., 2019).

Compared with other beaches on the east coast of the United States, Gulf of Mexico beaches have minimal dunes and higher amounts of vegetation due to the smaller amount of wave energy that the Gulf produces. This contrasts with beaches on the Atlantic coast, which have high dunes and less vegetation from the intense wave energy and storms arising from the Atlantic Ocean (K. Bergman, personal communication). Few studies have explored plant penetration into sea turtle nests on the Gulf of Mexico (Shaver et al., 2020) and none have compared species of plants that impact nests on Florida Gulf of Mexico beaches.

As most loggerhead nesting occurs on the Atlantic coast of Florida, few studies focus on the Gulf of Mexico nesting population and less is known about the detrimental impacts on nesting in the region. The goal of this project was to determine how plant roots impact loggerhead sea turtle clutch success on an important sea turtle rookery in the Gulf of Mexico. We hypothesized that plants negatively impact hatch and emergence success, but due to the nature of these beaches, invasive plants have a greater impact on sea turtle clutches than native plants. This region has been monitored for over four decades and we modeled which factors affect plant invasion of the nest chamber, including determining the distance from the dune at which roots will penetrate turtle nest chambers.

## 2 METHODS

### 2.1 Study site

**FIGURE 3:**
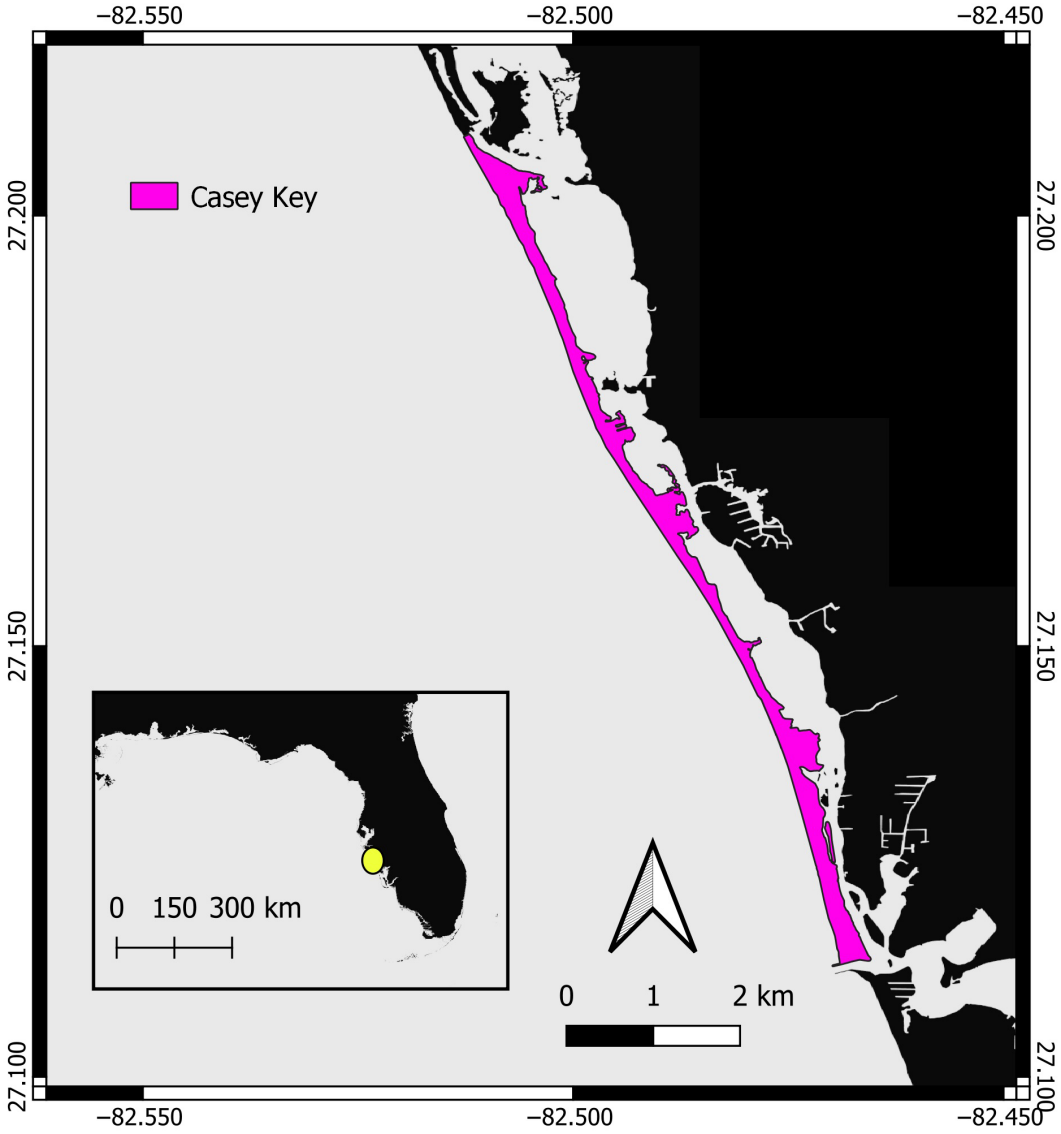
Map of Casey Key, Sarasota County, Florida, USA.

This study was conducted on Casey Key in Sarasota County, Florida, USA. Casey Key is a 7.3‐mile‐long barrier island on the Gulf of Mexico and is part of the largest loggerhead rookery in the Gulf of Mexico (Figure 3) (Lasala et al., 2023). Casey Key contains numerous large single‐family homes, which are protected by the dune system that runs the length of the island, broken up in some areas by seawalls and protective sandbags.

### 2.2 Data collection

Annually, during the sea turtle nesting season (April 15 to October 31), staff from Mote Marine Laboratory's Sea Turtle Conservation and Research Program (STCRP) patrol beaches in the Sarasota region to identify marine turtle nesting behavior. When a nest is found, STCRP staff determine the species of marine turtle from their crawl patterns. STCRP staff measure the distance between the nest and the high water mark (m) and the distance from the nest to the dune or barrier (m; e.g., seawalls). These measurements are later used to determine the portion of the beach in which the nest was constructed (lower, middle, or upper). A GPS reading of the nest location is also taken (starting in 2004). All monitored nests are staked off and will be observed daily for activity, predation, and hatchling emergence. Three days following hatchling emergence or after 70 days of no activity, the nest is excavated, and the contents are quantified. Loggerhead nest data were assessed in two ways for this project: a case study conducted in 2022 and a model of long‐term data.

The first dataset focused on identifying plant species during nest excavations on Casey Key between July and August of 2022. All activities were permitted under Florida Fish and Wildlife Conservation Commission (FWC) Marine Turtle Permit 216. Although all excavations from 1987 to 2022 included instructions to identify if plants were present, plant species were not identified. This case study specifically identified what plants were present near the nest and in the nest. A subset of monitored nests from 2022 were included in this case study. During nest excavation, the contents of the chamber were carefully removed and separated between hatched eggs, unhatched eggs, live and dead pipped hatchlings, live and dead hatchlings, and root encased and root invaded eggs. Pipped refers to eggs where the hatchling broke through the shell, but did not fully hatch from the egg. Multiple measurements using a flexible measuring tape were taken, including nest chamber width, the surface to egg depth, and the surface to bottom depth. Live hatchlings and live pipped hatchlings were taken to Mote Marine Laboratory's sea turtle hospital for care or were released at the site of collection if possible. Following excavation, nearby dune plants were recorded and identified. Observations were made of the nest chamber to confirm root penetration, and, where possible, roots were followed to the originating plant to determine which species were present within the chamber. Eggs were carefully observed to determine whether roots encased (surrounded) or invaded (went through) the eggs. Eggs that displayed root damage were separated between hatched and unhatched and then all nest contents were quantified.

The second dataset focused on excavation data collected by STCRP from 1987 to 2022 for all nests monitored on Casey Key, Sarasota County, Florida during that time frame (FWC Marine Turtle Permit 048, FWC Marine Turtle Permit 216, and Consent Permit Number FWC RP #915). Monitoring schemas were modified in 2003, 2013, and 2022 (see Lasala et al., 2023 for more details), and raw data needed to be checked for irregularities.

From these raw data from both datasets, we calculated internal nest chamber depth (top minus bottom), incubation length (emergence date minus date clutch was laid), total eggs, the proportion of root‐damaged eggs to the total number of eggs, and hatch success and emergence success. Hatch success provided a proportion of the number of eggs that hatched (Equation 1) and emergence success determined the proportion of the hatchlings that emerged from the nest (Equation 2).

(1) $$\text{Hatch success}\%=\left(\frac{\text{Hatched eggs}}{\text{Total eggs}}\right)*100$$

(2) $$\begin{array}{c} \text{Emergence success}\%\\ =\left(\frac{\left(\text{Hatched eggs}-\text{Dead hatchlings}-\text{Live hatchlings}\right)}{\text{Total eggs}}\right)*100 \end{array}$$

Data from these two datasets were analyzed separately. While there were ≥5000 observations in the long‐term dataset, identification of plant intrusion was inconsistent by year. Furthermore, prior to 2022, plant species were not identified, and thus a broader model must be assessed. All data were assessed using Program R (R Core Team, 2022) and visualized using the package *ggplot2* (Wickham, 2016). Both datasets were tested for normality: The case study data were assessed using a Shapiro–Wilk's test and the long‐term dataset was assessed using a Kolmogorov–Smirnov test due to the large number of observations (≥5000). Most biological data deviated from a normal distribution and thus we used non‐parametric (rank‐based) models.

For the case study, Kruskal–Wallis tests were used to determine whether there was a difference in hatch success and emergence success due to the different plant types. Independent Mann Whitney *U*‐tests were used to determine whether there were differences in hatch and emergence success between the top two plant species that impacted the most nests.

For the longitudinal dataset, generalized linear models (GLM) were run and assessed for best fit using AIC values (Table 1). Depth to bottom, cavity width, root damage proportion, distance to mean high water line (m), total eggs destroyed, and distance to barrier (m) were the independent variables (*x*). These independent variables were tested against hatch and emergence success (*y*_1_ and *y*_2_). Post hoc Dunn tests were then run to determine which categorical variables had a significant impact on hatch success and emergence success. Kruskal–Wallis tests were used to determine whether nest placement on the beach had a significant impact on root damage and root invasion of nests. Due to data nonlinearity, a generalized additive model (GAM) was run using the package *mgcv* (Wood, 2004) to determine if the number of nests have increased over time. Finally, a Spearman's rank correlation was run to determine whether the number of nests impacted by root invasion over time was correlated to the overall number of nests on Casey Key over the same time period.

**TABLE 1:** Top 5 AIC values for generalized linear models.

Model terms	∆AIC hatch success	Model terms	∆AIC emergence success
Depth to Bottom*Chamber Width*Root Damage Rate*Distance to Mean High Waterline (m)*Total Eggs Destroyed + Distance to Barrier (m)	0	Depth to Bottom*Chamber Width*Root Damage Rate*Distance to Mean High Waterline (m)*Total Eggs Destroyed + Distance to Barrier (m)	0
Depth to Bottom*Total Eggs Destroyed*Distance to Barrier (m)*Chamber Width*Root Damage Rate + Distance to Mean High Waterline (m)	2.19	Depth to Bottom*Total Eggs Destroyed*Distance to Barrier (m)*Chamber Width*Root Damage Rate + Distance to Mean High Waterline (m)	1.91
Depth to Bottom*Distance to Mean High Waterline (m)*Total Eggs Destroyed*Distance to Barrier (m)*Chamber Width + Root Damage Rate	9.28	Depth to Bottom*Total Eggs Destroyed*Distance to Barrier (m)*Chamber Width + Root Damage Rate + Distance to Mean High Waterline (m)	7.82
Depth to Bottom*Total Eggs Destroyed*Distance to Barrier (m)*Chamber Width + Root Damage Rate + Distance to Mean High Waterline (m)	12.36	Depth to Bottom*Chamber Width*Root Damage Rate + Distance to Mean High Waterline (m) + Total Eggs Destroyed + Distance to Barrier (m)	15.99
Depth to Bottom*Chamber Width*Root Damage Rate + Distance to Mean High Waterline (m) + Total Eggs Destroyed + Distance to Barrier (m)	24.72	Depth to Bottom*Total Eggs Destroyed*Distance to Barrier (m)*Chamber Width*Root Damage Rate*Distance to Mean High Waterline (m)	18.42

## 3 RESULTS

### 3.1 Case study

**FIGURE 4:**
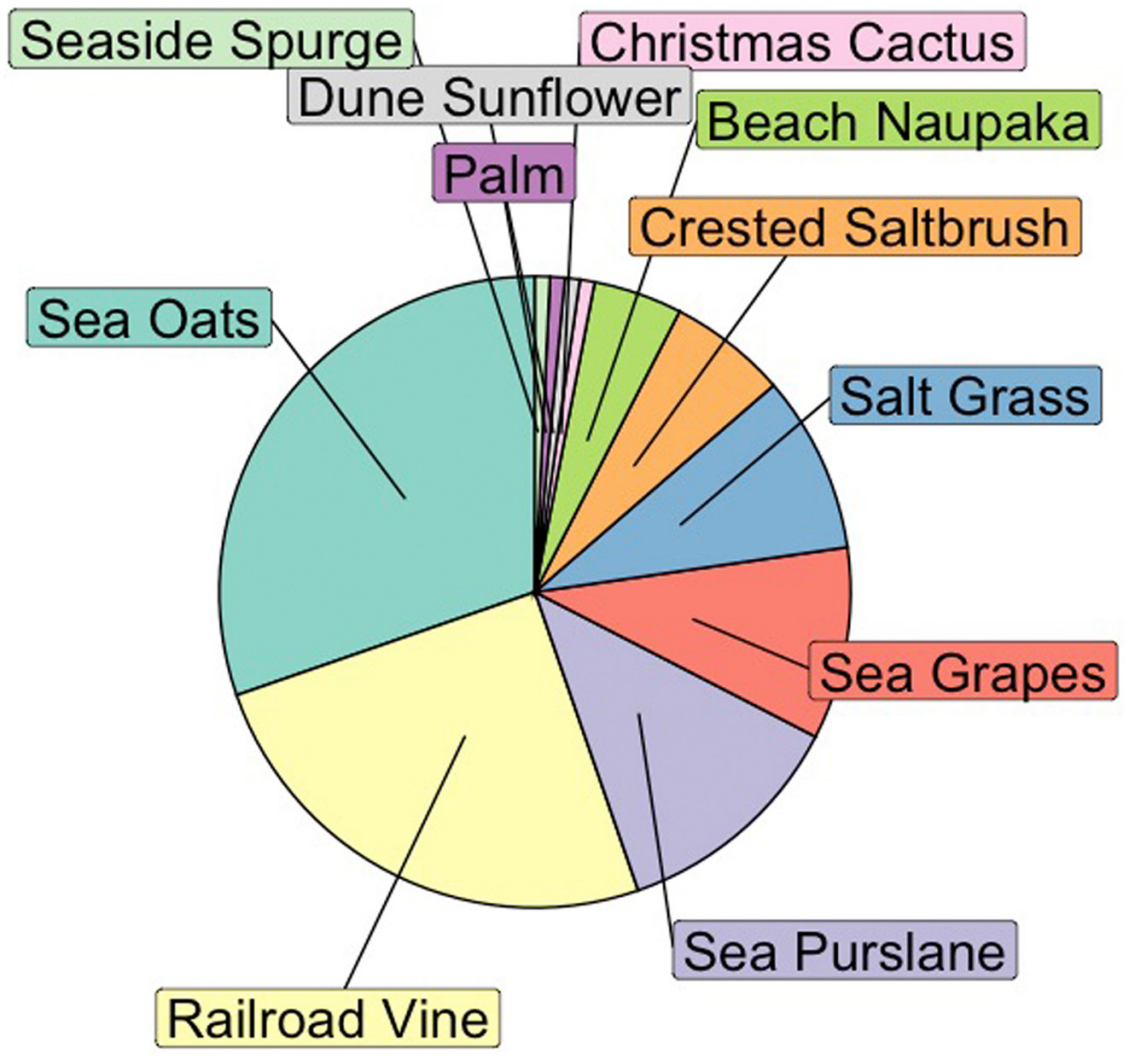
Represents the plants that were observed near nests in the case study. The size of the “pieces” represents the number of times the plants were observed.

Eleven species of vegetation were found nearby loggerhead nests excavated on Casey Key in 2022 (Figure 4). However, of these, only three types of vegetative roots were found within the nest chamber: sea oats (*Uniola paniculata*), railroad vine (*Ipomoea pes‐caprae*), and sea purslane (*Sesuvium portulacastrum*).

**FIGURE 5:**
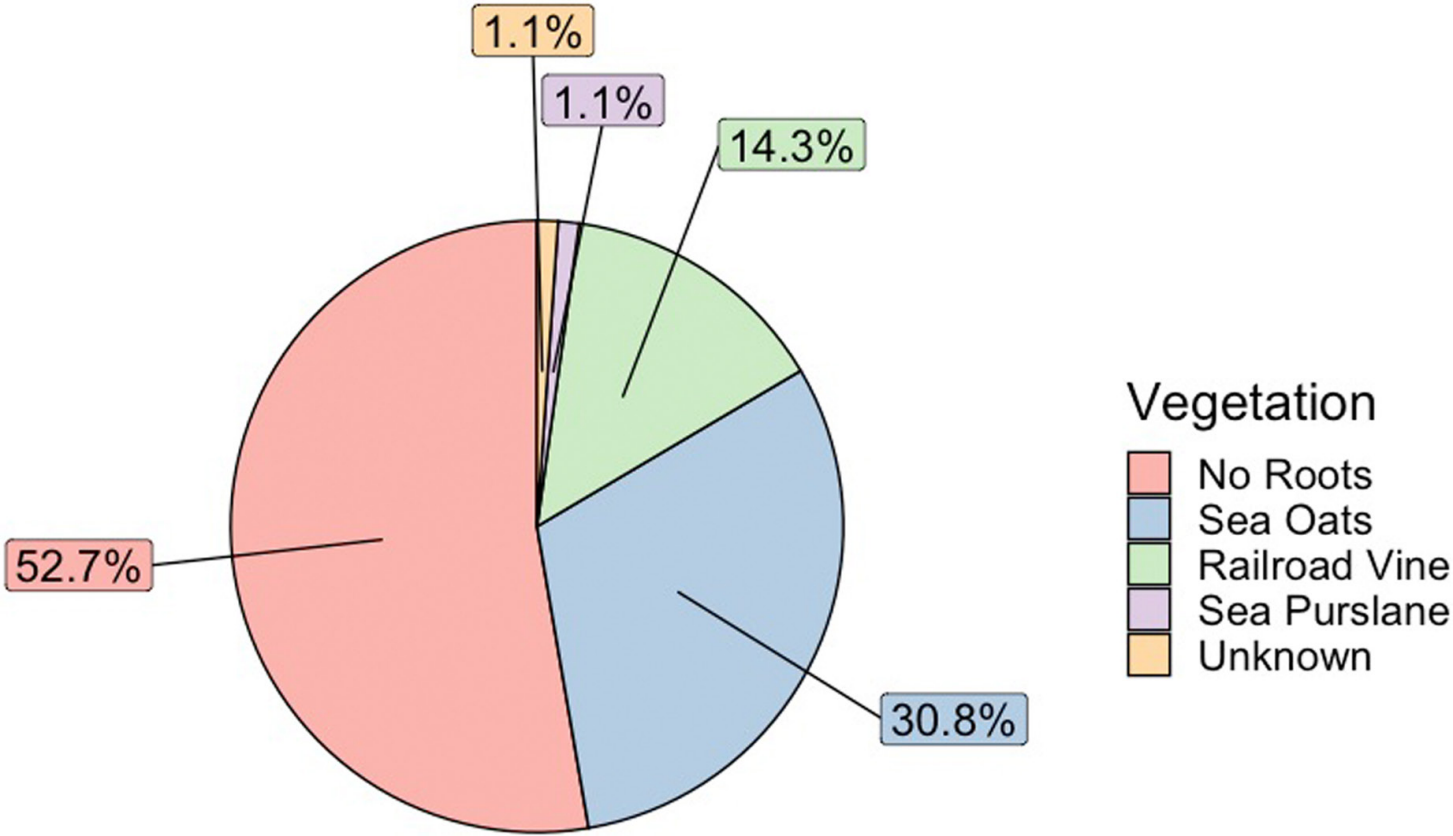
Plant species found in case study nests and their respective proportions. 52.7% of case study nests were observed to have no roots in the nest chamber, while 47.3% were observed to have roots in the nest chamber.

Two of the three types of vegetation affecting nests excavated in the 2022 case study (Figure 5) were found in a high number of nests: sea oats were found in 28 nests (30.8%), railroad vine in 13 nests (14.3%), and sea purslane in one nest (1.1%). Of the 91 nests excavated for this study, 48 (53%) did not have roots within the nest chamber. For nest cavities impacted by sea oat roots, the average hatch success was 77.9% and for nest cavities impacted by railroad vine hatch success was 70.7%; however, this difference was not significant (*W* = 168.5, *p* = .378). Similarly, the average emergence success was 76.3% and 69.8%, respectively; but again, this difference was not significant (*W* = 184, *p* = .632). The average distance from the dune plants to nests found with roots in them was 0.767 m for sea oats and 4.05 m for railroad vine.

### 3.2 Long‐term data

**FIGURE 6:**
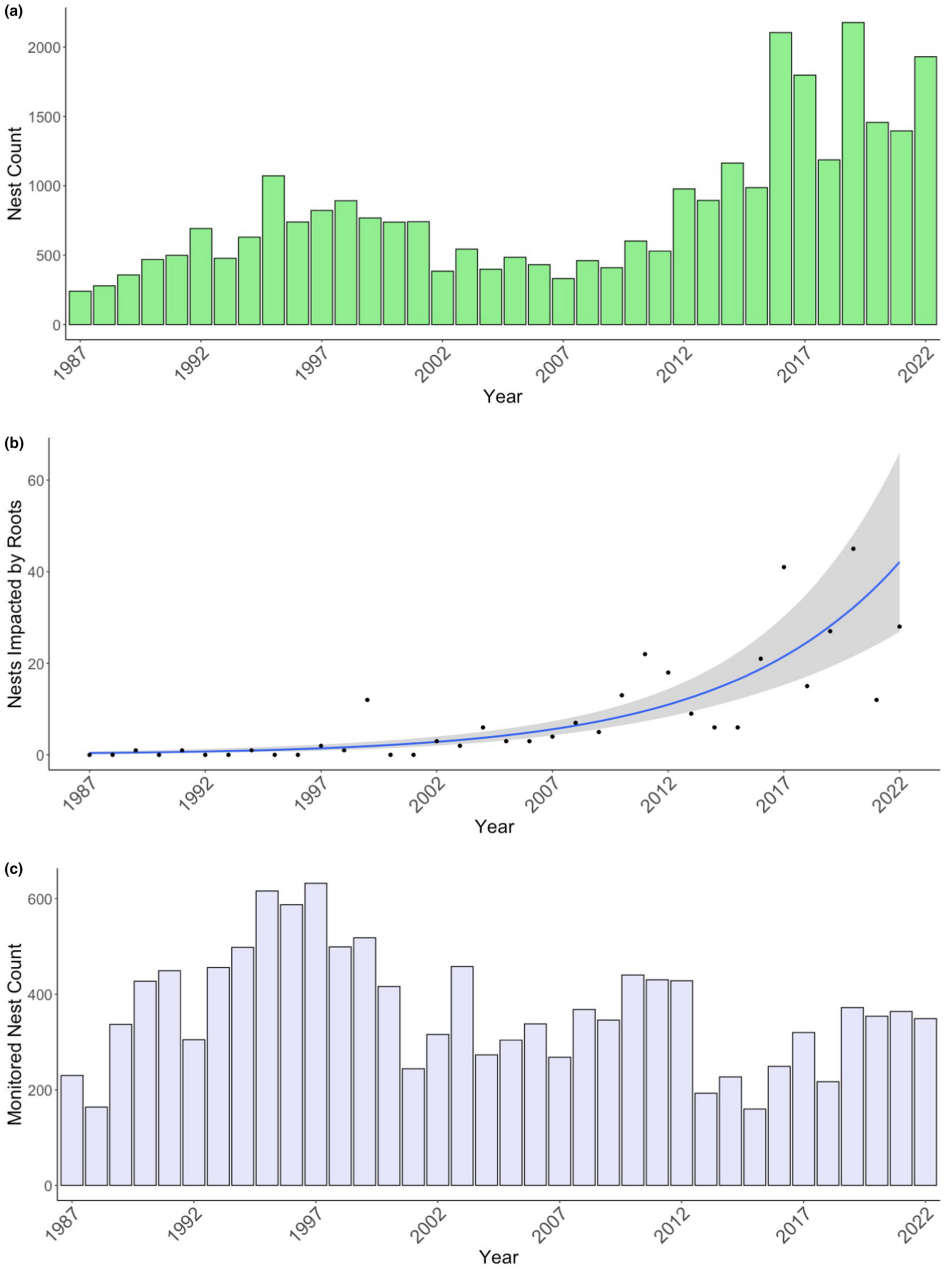
(a) Total number of loggerhead sea turtle nests on Casey Key from 1987 to 2022. (b) The sum of nests impacted by roots each year from 1987 to 2022, note the trend line (quadratic) and the standard error (gray shading). (c) Monitored nest counts on Casey Key from 1987 to 2022.

In this region of Florida, loggerhead nest counts have steadily increased since 2008 (Lasala et al., 2023, Figure 6a) and the number of nests with root presence have also steadily increased. Over the study period, 2.4% (*n* = 302) of 13,152 loggerhead nests laid on Casey Key were impacted by roots. The number of nests invaded by roots has increased significantly over time (*F* = 21.28, *p* < .001, *R* ^2^ = .625) (Figure 6b). The number of nests impacted by roots are positively correlated with the number of monitored nests on Casey Key (*S* = 3191.8, *p* < .001, ρ = 0.589) (Figure 6c). For this dataset, the best fitting GLM variables are reported in Table 1.

**FIGURE 7:**
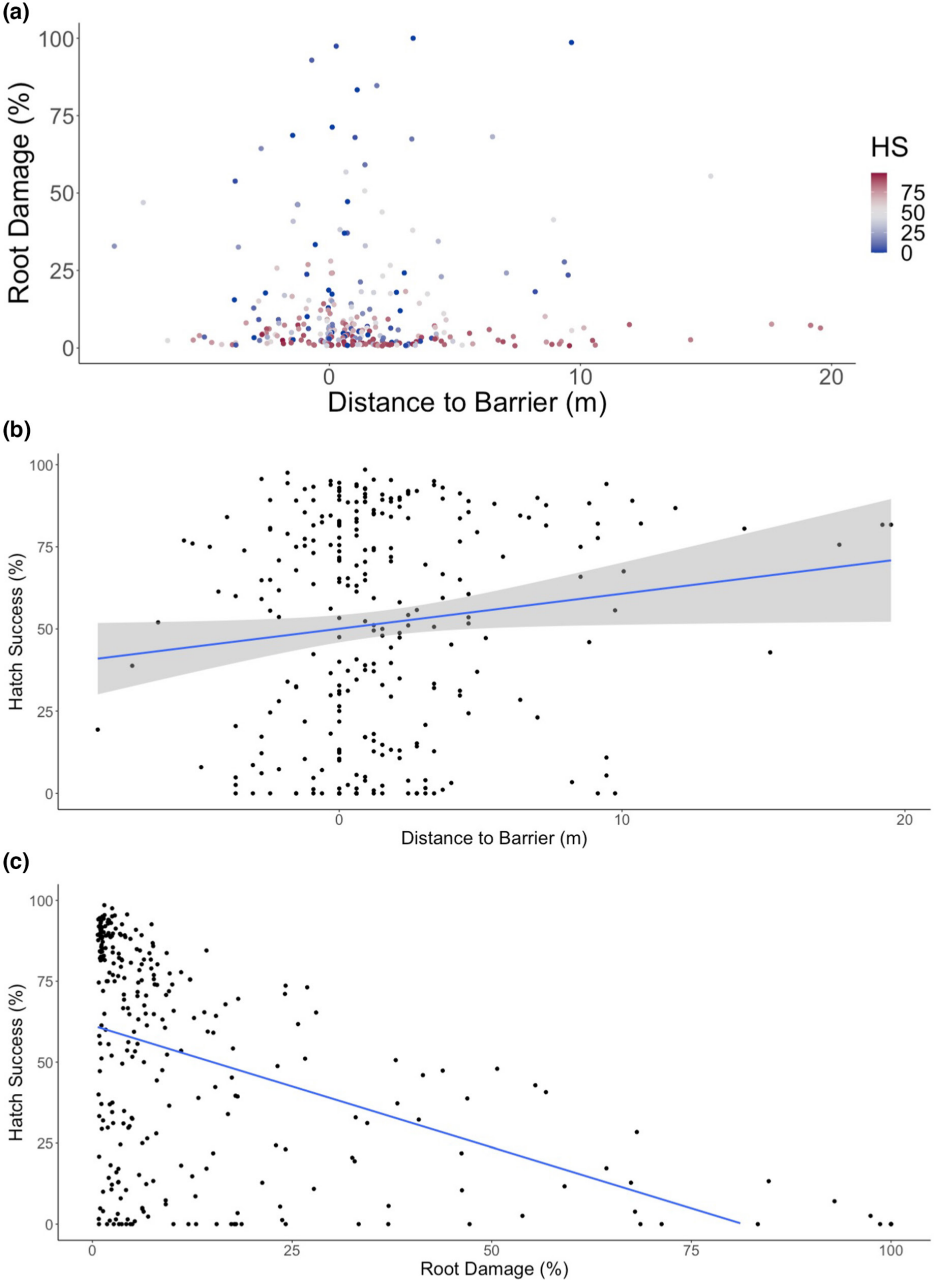
(a) Distance to the barrier (m) and proportion of root damage, colored by hatch success (HS%) of nests excavated between 2003 and 2022. (b) Distance to the barrier (m) and hatch success (%) of nests excavated between 2003 and 2022. (c) Proportion of root damage and hatch success (%) of nests excavated between 2003 and 2022.

72% of the 8749 nests that had data for their location on the beach were located within the upper third of the beach, closer to the barrier (dune). However, nests had a lower proportion of root damage and a higher hatch and emergence success if they were farther from the barrier, but not below the high tide line (HS: *t* = −6.198, *p* < .001; ES: *t* = −6.477, *p* < .001, *R* ^2^ = .015). The average hatch success for nests without root presence was 72.3% and with roots was 51.4%; for emergence success, the averages were 68.2% and 50%, respectively. Nests with a higher proportion of root damage had a lower proportion of hatch success, and this trend was similar for emergence success (Figure 7).

**FIGURE 8:**
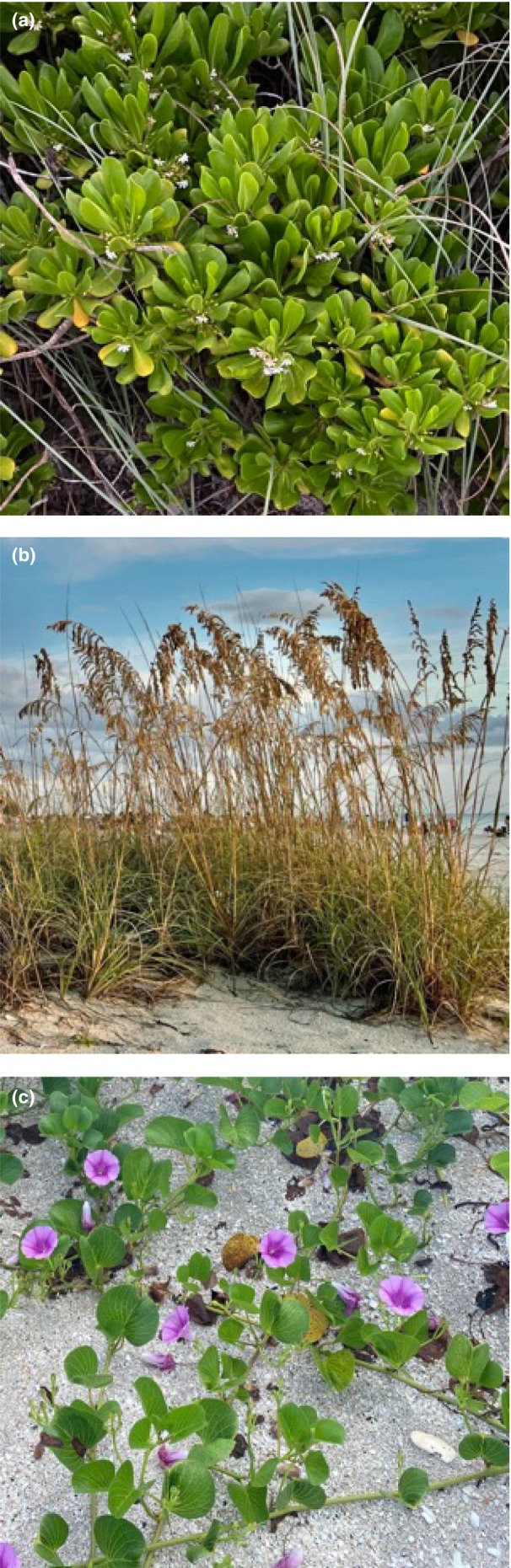
Plant species found nearby (a) and within (b/c) loggerhead sea turtle nests on Casey Key, FL during the 2022 case study. (a) Beach naupaka. (b) Sea oats. (c) Railroad vine.

In 2022, no invasive plants were found in the dune (barrier) and only one type of non‐native plant was found: beach naupaka (*Scaevola taccada*, Figure 8a). No invasive or non‐native plants were found within the nest chamber of any nests excavated during the case study of 2022. Sea oats and railroad vine (Figure 8b,c), both native plants, were found in nests and decreased success of both hatching and emergence.

## 4 DISCUSSION

This study represents the first investigation of the impact of dune plants on sea turtle nests on Florida's Gulf of Mexico beaches. We discovered that root presence in the nest chamber decreases both hatch and emergence success of hatchlings. A further assessment determined that nests laid closer to the dune are more likely to have a higher proportion of root damage and thus a lower hatch and emergence success.

To our knowledge, only three previously published studies have identified that plants can have impact sea turtle nests. Shaver et al. (2020) noted that 0.7% of Kemp's ridley (*Lepidochelys kempii*) nests laid on the Texas Gulf of Mexico coastline were impacted by plant penetration, but they did not identify the types of plants present in nests. In St. Croix, US Virgin Islands, Conrad et al. (2011) found leatherback (*Dermochelys coriacea*) hatchlings trapped by roots in 40% of their experimental nests and determined that the vegetation on the dunes and the beach limited the space available for nesting. The authors determined that when a leatherback egg was encased or invaded, development was arrested. Contrary to this, our findings show that loggerhead hatchlings can emerge from eggs that are encased or invaded by roots. Furthermore, the prevailing species of plant in St. Croix was railroad vine, whereas sea oats were more prevalent on Casey Key. It is likely not only that plant species impact the nests differently but also that sea turtle species hardiness has an impact on hatchling success when stressed by root invasion.

Hannan et al. (2007) also found that plant roots penetrated loggerhead and green sea turtle (*Chelonia mydas*) eggs, with a higher percentage of penetration in loggerhead eggs. Isotopic and nitrogen concentration analysis was conducted on the eggs and nearby sea oats to determine the contribution of egg nutrients to the dune vegetation. They found a positive correlation between nitrogen signatures in sea oats and nest density, suggesting that plant roots facilitate nutrient transfer between eggs and the dune environment. On Florida's Atlantic coast, Osegovic (2001) found that green sea turtle nests were less impacted plant roots than loggerhead nests. Osegovic also found a lower rate of intrusion for loggerhead nests than our study (12.5% of nests excavated) and only 0.26% of eggs were directly impacted by root predation. Sea grapes and sea oats were observed in this project, but egg penetration by roots was not segregated by species.

Sea oats are an essential native plant to Florida ecosystems due to their ability to retain sand and prevent dune erosion (Florida Statutes, 2022). Sea oats have extensive root systems that anchor them into the dune. Their primary root mass is concentrated in the upper 30 cm of the sand, which coincides with the depth from the surface to the first egg in loggerhead nest chambers (15 cm on average), while taproots extend deeper, into the internal chamber where eggs are located (to depths of 25 cm on average) (Hannan et al., 2007). Sea oats also have a symbiotic relationship with nitrogen fixing bacteria, which converts nitrogen in the sand into a form that is usable to the plant for growth (Will & Sylvia, 1990). Sea oats are protected by state law (Florida Wildlife Federation, 2020), and they grow more vigorously in areas that are well fertilized (Baker & Dahl, 1981), possibly explaining their root presence in sea turtle nests. A proposed management strategy could be to fertilize beaches enough that sea oats do not need to seek out additional nutrients from the sea turtle eggs. However, fertilization could reduce plant root biomass (Aerts et al., 1991) and thus compromise dune stabilization by roots. Moreover, nitrogen fertilization raises the probability of nutrient runoff and coastal eutrophication (Howarth & Marino, 2006). Negative impacts on eggs are also a possibility; in a study conducted on common snapping turtle eggs (*Chelydra serpentina*), ammonia was found to be acutely toxic, impacting embryo development and decreasing hatch success by 100% when application rates were over 5.5 times the recommended amount (de Solla et al., 2011).

In addition to sea oats, the other most common plant found in loggerhead nests was railroad vine. Railroad vine is native to dune habitats along tropical and subtropical coasts in the Gulf of Mexico and the Northwest Atlantic Ocean (Brown & Hazell, 2010). Due to their crawling growth form, these vines spread down beaches, as much as 9 m in width in all directions and penetrate up to 90 cm deep into the sand (Brown & Hazell, 2010). Railroad vine is often used in beach restoration and stabilization and is a primary colonizer of beaches due to its ability to grow quickly and thrive in sandy, nutrient‐poor environments (Brown & Frank, 2020). It also provides habitat for dune species, including those that are threatened or endangered (Brown & Hazell, 2010). Railroad vine was found to impact sea turtle nests in the Caribbean (Conrad et al., 2011), but our study is the only other to describe its impact on sea turtles. Sea purslane was also found as a root in one of the nests in the case study; it often acts as an important pioneer species in dune ecosystems, colonizing the nutrient‐poor environment before most other species and preparing the substrate for other plant species (Lonard & Judd, 1997).

Sea oats and railroad vine are native plants, but it is unknown if they physically seek excess nutrients in their ecosystem from sea turtle eggs. Future studies should determine whether plant roots actively seek out the nutrients inside the sea turtle eggs (i.e., actively growing towards them) or if this overlap and its benefits for dune vegetation are coincidental. Regardless, the plants that find the excess nutrients that the sea turtle eggs provide will thrive in the otherwise nutrient‐poor environment, while those that do not may grow as extensively.

The number of nests laid on Casey Key, Florida has increased steadily since 2008. Lasala and colleagues (2023) posited that this growth is due to the use of turtle excluder devices in the Gulf of Mexico that has increased sea turtle survivability by reducing fisheries bycatch (Gallaway et al., 2016). Furthermore, the Endangered Species Act of 1973 increased protections to the turtles and their habitats in the USA, and regional managers have increased actions to conserve these species (United States, 1983). Coupled to these increasing nest numbers, our study showed an increase in the number of nests impacted by roots annually. This increase may be due to successful dune restoration efforts by beach managers that have increased total dune plant biomass over time by planting more vegetation (Martínez et al., 2013). STCRP does not monitor every nest on the beach and so it is not known whether the increasing number of nests has created more opportunities for growing plant roots to coincide with nests.

Nest placement on the beach also plays a large role in how at‐risk nests are of being invaded. Nests laid closer to the dune have a higher risk of invasion and damage by plant roots but are more likely to survive storms and high water events (Gravelle & Wyneken, 2022). Loggerhead sea turtles on the Gulf coast of Florida prefer to lay their nests farther from the tide line and closer to the dune; however, they are more successful at nesting in the middle of the beach (Lasala et al., 2023). Nesting females may be deterred from nesting directly in a vegetated area due to the increased difficulty in digging the nest chamber. However, if there are no roots in a specific area when the female is nesting, there is no deterrence from nesting in that spot and no indication that this nest site will not be suitable for a different turtle later in the season. Future studies should quantify if plants affect green sea turtle nests differently, as this species tends to nest far closer to the dune more often than the loggerheads (Lasala et al., 2023). Green sea turtles also tend to create larger body pits than loggerheads and may have less root invasion if they uproot the majority of vegetation surrounding their nest chamber. Casey Key, Florida is a very flat beach with no high dunes; vegetation grows at the barrier between the beach and human development, but there is not a high sand dune. Future studies should examine beaches with different dune elevations, as steeper, vegetated dunes may not have the same root expanse outward as observed on Casey Key and may exhibit more vertical root growth into the dune.

A limitation of this study was the inconsistent collection and reporting of root data during excavations from 1987 to 2002. Further analysis is also needed to understand the full extent of root impact leading up to the present day. These limitations notwithstanding, findings from this study suggest that root–turtle interactions may be important to populations of dune plants and beach‐nesting turtles.

## 5 CONCLUSIONS AND FUTURE DIRECTIONS

Sea turtles are negatively impacted by the presence of dune plant roots within their nest chamber. While we found that plant roots have a negative impact on loggerhead sea turtle nest success on Casey Key, Florida. We did not find evidence for whether invasive plants had disproportionate impacts compared with native plants due to the lack of invasive plants at the study site. More research is needed to determine whether other beaches have strong interactions between plant root invasion and sea turtle nests, and if invasive plant impacts differ from those of native plants. More research is also needed to establish whether some nesting areas are more at risk of plant root invasion than others, especially in relation to coastline dynamics and dune slope. Lastly, future studies should explore why root invasion is increasing over time and the implications of this trend for the sea turtle and dune plant population dynamics.

## AUTHOR CONTRIBUTIONS

**Olivia T. Redding:** Conceptualization (lead); data curation (lead); formal analysis (lead); investigation (lead); methodology (lead); resources (lead); software (lead); validation (lead); visualization (lead); writing – original draft (lead); writing – review and editing (lead). **Max C. N. Castorani:** Formal analysis (supporting); resources (supporting); software (supporting); supervision (supporting); validation (supporting); visualization (supporting); writing – original draft (supporting); writing – review and editing (supporting). **Jake Lasala:** Conceptualization (supporting); data curation (supporting); formal analysis (supporting); investigation (supporting); methodology (supporting); resources (supporting); software (supporting); supervision (lead); validation (supporting); visualization (supporting); writing – original draft (supporting); writing – review and editing (supporting).

## CONFLICT OF INTEREST STATEMENT

All authors declared that they have no competing interests.

## Data Availability

Case study data has been uploaded to DRYADS and can be accessed using the following link: https://datadryad.org/stash/share/M5UZt5fqc9SW1oR86JVHs75yYuUvohUIs6g8TlcSA0o. Long‐term data is available if requested from J. Lasala.

## References

[ece311207-bib-0001] Ackerman, R. A. (1997). The nest environment and the embryonic development of sea turtles. In P. Lutz , & J. Musick (Eds.), The biology of sea turtles (pp. 83–106). CRC Press.

[ece311207-bib-0002] Aerts, R. , Boot, R. G. A. , & van der Aart, P. J. M. (1991). The relation between above‐ and belowground biomass allocation patterns and competitive ability. Oecologia, 87, 551–559. 28313698 10.1007/BF00320419

[ece311207-bib-0003] Baker, R. L. , & Dahl, B. E. (1981). Determining vigor of natural and planted strands of sea oats on the Texas Gulf Coast. The Southwestern Naturalist, 26(2), 117–123.

[ece311207-bib-0004] Bolten, A. B. , & Witherington, B. E. (2003). Loggerhead sea turtles (p. 111, 114–117, 133, 244–246, 304). Smithsonian Institution.

[ece311207-bib-0005] Bouchard, S. S. , & Bjorndal, K. A. (2000). Sea turtles as biological transporters of nutrients and energy from marine to terrestrial ecosystems. Ecology, 81(8), 2305–2313.

[ece311207-bib-0006] Brown, S. H. , & Frank, M. S. (2020). Railroad vine (Ipomoea pes‐caprae): Identification and uses. University of Florida.

[ece311207-bib-0007] Brown, S. H. , & Hazell, J. (2010). Railroad Vine: Ipomoea pes‐caprae, a Florida Native Plant. University of Florida.

[ece311207-bib-0008] Cardinale, B. , Primack, R. , & Murdoch, J. (2019). Conservation biology. Oxford University Press Academic US. https://online.vitalsource.com/books/9781605358826

[ece311207-bib-0009] Charbonneau, B. R. , Wnek, J. P. , Langley, J. A. , Lee, G. , & Balsamo, R. A. (2016). Above vs. belowground plant biomass along a barrier Island: Implications for dune stabilization. Journal of Environmental Management, 182, 126–133. 27459337 10.1016/j.jenvman.2016.06.032

[ece311207-bib-0010] Conrad, J. R. , Wyneken, J. , Garner, J. A. , & Garner, S. (2011). Experimental study of vegetation impact and control on leatherback sea turtle *Dermochelys coriacea* nests. Endangered Species Research, 15, 13–27. 10.3354/esr00361

[ece311207-bib-0011] Costa, L. , Rangel, D. F. , & Zalmon, I. (2023). Effect of touristic activities on seabirds' habitat selection on sandy beaches. Oceanological and Hydrobiological Studies, 52(3), 287–298.

[ece311207-bib-0012] de Solla, S. R. , Martin, P. A. , & Mikoda, P. (2011). Toxicity of pesticide and fertilizer mixtures simulating corn production to eggs of snapping turtles (*Chelydra serpentina*). Science of the Total Environment, 409(20), 4306–4311. 21831407 10.1016/j.scitotenv.2011.06.046

[ece311207-bib-0013] Ewel, J. J. , O'Dowd, D. J. , Bergelson, J. , Daehler, C. C. , D'Antonio, C. M. , Gomez, L. D. , Gordon, D. R. , Hobbs, R. J. , Holt, A. , Hopper, K. R. , Hughes, C. E. , LaHart, M. , Leakey, R. R. B. , Lee, W. G. , Loope, L. L. , Lorence, D. H. , Louda, S. M. , Lugo, A. E. , McEvoy, P. B. , … Vitousek, P. M. (1999). Deliberate introductions of species: Research needs. Bioscience, 49, 619–630.

[ece311207-bib-0014] Florida Department of Environmental Protection (FDEP) . (2024). Beaches . Retrieved 2024, from https://floridadep.gov/rcp/beaches

[ece311207-bib-0015] Florida Fish and Wildlife Conservation Commission (FWC) . (2022). Index nesting beach survey totals (1989–2021) . Retrieved 2022, from https://myfwc.com/research/wildlife/sea‐turtles/nesting/beach‐survey‐totals/

[ece311207-bib-0016] Florida Statutes . (2022). Justia US law . Retrieved from https://law.justia.com/codes/florida/2022/title‐xi/chapter‐161/part‐i/section‐161‐242/

[ece311207-bib-0017] Florida Wildlife Federation . (2020). Sea oats . Retrieved from https://floridawildlifefederation.org/sea‐oats/

[ece311207-bib-0018] Gallaway, B. J. , Gazey, W. J. , & Cole, J. G. (2016). An updated description of the benefits and consequences of red snapper shrimp trawl bycatch management actions in the Gulf of Mexico. North American Journal of Fisheries Management, 2, 414–419.

[ece311207-bib-0019] Gravelle, J. , & Wyneken, J. (2022). Resilient eggs: Highly successful Loggerhead Sea turtle nesting sites vary in their characteristics. Frontiers in Ecology and Evolution, 10, 853835.

[ece311207-bib-0020] Guerra, C. C. , Ricardo, J. A. , Ávila, R. B. , Bretos, F. , & Álvarez, P. P. (2021). Influence of sandy coast vegetation on the reproductive success of green turtles at Cuban nesting beaches. Chelonian Conservation and Biology: Celebrating 25 Years as the World's Turtle and Tortoise Journal, 20(2), 254–264.

[ece311207-bib-0021] Hannan, L. B. , Roth, J. D. , Ehrhart, L. M. , & Weishampel, J. (2007). Dune vegetation fertilization by Nesting Sea turtles. Ecology, 88(4), 1053–1058. 17536720 10.1890/06-0629

[ece311207-bib-0022] Hiatt, D. , Serbesoff‐King, K. , Lieurance, D. , Gordon, D. R. , & Luke Flory, S. (2019). Allocation of invasive plant management expenditures for conservation: Lessons from Florida, USA. Conservation Science and Practice, 1(7), e51. 10.1111/csp2.51

[ece311207-bib-0023] Howarth, R. , & Marino, R. (2006). Nitrogen as the limiting nutrient for eutrophication in coastal marine ecosystems: Evolving views over three decades. Limnology and Oceanography, 51(1 Part 2), 364–376.

[ece311207-bib-0024] IUCN Red List . (Accessed 2022). Retrieved from https://www.iucnredlist.org/

[ece311207-bib-0025] Lasala, J. A. , Macksey, M. C. , Mazzarella, K. T. , Main, K. L. , Foote, J. J. , & Tucker, A. D. (2023). Forty years of monitoring increasing sea turtle relative abundance in the Gulf of Mexico. Scientific Reports, 13, 17213. 10.1038/s41598-023-43651-4 37821522 PMC10567714

[ece311207-bib-0026] Lonard, R. I. , & Judd, F. W. (1997). The biological Flora of coastal dunes and wetlands. *Sesuvium portulacastrum* . Journal of Coastal Research, 13(1), 96–104.

[ece311207-bib-0027] Martínez, M. L. , Gallego‐Fernández, J. B. , & Hesp, P. A. (2013). Restoration of coastal dunes (p. 344). Springer Series on Environmental Management, Springer‐Verlag Berlin Heidelberg.

[ece311207-bib-0028] Osegovic, K. (2001). Hatching success, embryonic mortality, and infertility in loggerhead (*Caretta caretta*) and green (*Chelonia mydas*) sea turtles nesting in Brevard County, Florida. Retrospective Theses and Dissertations, 1306. https://stars.library.ucf.edu/rtd/1306

[ece311207-bib-0029] Pyšek, P. , Jarošík, V. , Hulme, P. E. , Pergl, J. , Hejda, M. , Schaffner, U. , & Vilà, M. (2012). A global assessment of invasive plant impacts on resident species, communities and ecosystems: The interaction of impact measures, invading species' traits and environment. Global Change Biology, 18, 1725–1737.

[ece311207-bib-0030] R Core Team . (2022). R: A language and environment for statistical computing. R Foundation for Statistical Computing. https://www.R‐project.org/

[ece311207-bib-0031] Richardson, R. B. , & Nicholls, S. (2021). Characterizing the cultural ecosystem services of coastal sand dunes. Journal of Great Lakes Research, 47(2), 546–551.

[ece311207-bib-0032] Schlacher, T. A. , De Jager, R. , & Nielsen, T. (2011). Vegetation and ghost crabs in coastal dunes as indicators of putative stressors from tourism. Ecological Indicators, 11, 284–294. 10.1016/j.ecolind.2010.05.006

[ece311207-bib-0033] Shaver, D. J. , Frandsen, H. R. , Shelby Walker, J. , George, J. A. , & Gredzens, C. (2020). Threats to Kemp's ridley sea turtle (*Lepidochelys kempii* Garman, 1880) nests incubating *in situ* on the Texas coast. Herpetology Notes, 13, 907–923.

[ece311207-bib-0034] Sigren, J. M. , Figlus, J. , & Armitage, A. R. (2014). Coastal sand dunes and dune vegetation: Restoration, erosion, and storm protection. Shore & Beach, 82(4), 5–12.

[ece311207-bib-0035] Staines, M. N. , Booth, D. T. , & Limpus, C. J. (2019). Microclimatic effects on the incubation success, hatchling morphology and locomotor performance in marine turtles. Acta Oecologica, 97, 49–56.

[ece311207-bib-0036] Stockton, R. (2023). Coastal research center . Retrieved February 15, 2023, from https://intraweb.stockton.edu/eyos/coastal/25yrConference/Beach‐Stabilization.pdf

[ece311207-bib-0037] Stoddard, M. A. , Miller, D. L. , Thetford, M. , & Branch, L. C. (2019). If you build it, will they come? Use of restored dunes by beach mice. Restoration Ecology, 27, 531–537. 10.1111/rec.12892

[ece311207-bib-0038] United States . (1983). The endangered species act as amended by public law 97–304 (the endangered species act amendments of 1982). U.S. G.P.O.

[ece311207-bib-0039] USFWS (US Fish and Wildlife Service) . (2017). Environmental conservation online system . Retrieved from http://ecos.fws.gov/ecp/

[ece311207-bib-0040] Wallace, B. P. , Sotherland, P. R. , Tomillo, P. S. , Bouchard, S. S. , Reina, R. D. , Spotila, J. R. , & Paladino, F. V. (2006). Egg components, egg size, and hatchling size in leatherback turtles. Comparative Biochemistry and Physiology Part A: Molecular & Integrative Physiology, 145(4), 524–532. 10.1016/j.cbpa.2006.08.040 17029994

[ece311207-bib-0041] Wickham, H. (2016). ggplot2: Elegant graphics for data analysis. Springer‐Verlag.

[ece311207-bib-0042] Will, M. E. , & Sylvia, D. M. (1990). Interaction of rhizosphere bacteria, fertilizer, and vesicular‐arbuscular mycorrhizal fungi with sea oats. American Society for Microbiology, 56(7), 2073–2079. 10.1128/aem.56.7.2073-2079.1990 PMC184562 16348236

[ece311207-bib-0043] Williams, M. J. (2007). Native plants for coastal restoration: What, when, and how for Florida (p. 51). USDA, NRCS, Brooksville Plant Materials Center.

[ece311207-bib-0044] Wood, S. N. (2004). Stable and efficient multiple smoothing parameter estimation for generalized additive models. Journal of the American Statistical Association, 99, 673–686.

